# Impacts of treatments on recurrence and 28-year survival of ischemic stroke patients

**DOI:** 10.1038/s41598-021-94757-6

**Published:** 2021-07-27

**Authors:** Ting-Ann Wang, Tzy-Haw Wu, Shin-Liang Pan, Hsiu-Hsi Chen, Sherry Yueh-Hsia Chiu

**Affiliations:** 1 Graduate Institute of Epidemiology and Preventive Medicine, College of Public Health, National Taiwan University, Taipei, Taiwan; 2 Division of Acute Infectious Diseases, Centers for Disease Control, Ministry of Health and Welfare, Taipei, Taiwan; 3 Department of Internal Medicine, National Taiwan University Hospital, Taipei, Taiwan; 4 Department of Physical Medicine and Rehabilitation, National Taiwan University Hospital, Taipei, Taiwan; 5 Department of Health Care Management, College of Management, and Healthy Aging Research Center, Chang Gung University, No. 259, Wenhua 1st Rd., Guishan Dist., Taoyuan City, 33302 Taiwan; 6 Division of Hepatogastroenterology, Department of Internal Medicine, Kaohsiung Chang Gung Memorial Hospital, Kaohsiung, Taiwan

**Keywords:** Cardiology, Health care, Medical research

## Abstract

Aspirin and nicametate are well-established therapies for preventing recurrence and mortality from stroke in patients diagnosed as ischemic stroke. However, their respective effects on the recurrence, making allowance for the duration of recurrence and death without the occurrence of recurrence, and long-term survival have not been well elucidated. We aimed to evaluate long-term effect of two kinds of treatment on cerebrovascular death among ischemic stroke patients with or without the recurrence of stroke. Data used in this study were derived from the cohort based on a multicenter randomized double-blind controlled trial during 1992 to 1995 with the enrollment of a total of 466 patients with first-time non-cardioembolic ischemic stroke who were randomly allocated to receive aspirin (n = 222) or nicametate (n = 244). The trial cohort was followed up over time to ascertain the date of recurrence within trial period and death until Sep of 2019. The time-dependent Cox regression model was used to estimate the long-term effects of two treatments on death from cerebrovascular disease with and without recurrence. A total of 49 patients experienced stroke recurrence and 89 cerebrovascular deaths was confirmed. Patients treated with nicametate were more likely, but non statistically significantly, to have recurrence (aHR: 1.73, 95% CI 0.96–3.13) as compared with those treated by aspirin. Nicametate reduced the risk of cerebrovascular death about 37% (aHR: 0.63, 95% CI 0.41–0.97) compared with aspirin. The aspirin group had a lower recurrence rate than the nicametate group even with recurrence after 1–2 years of follow-up of first stroke but the latter had significantly reduced death from cerebrovascular disease for nicametate group, which requires more research to verify.

## Introduction

Stroke is one of the main causes of death worldwide and the most common cause of disability^1^. Aspirin is well recommended for prevention of stroke and the risk of recurrent stroke^2–4^. The benefits of aspirin has been shown to decrease the stroke recurrence by 46–53%^5,6^ and all-cause mortality by 18%^7^. Stroke recurrence usually causes more serious brain damages and neurologic impairments, as well as increases mortality^8–11^. Patients who experience stroke recurrence were different from those who have a non-recurrence stroke in some clinical characteristics. Besides several factors associated with stroke outcomes that have already been identified^12–15^, most cause of deaths after stroke are association with cerebrovascular disease during early follow-up, but non-cerebrovascular deaths become increasingly leading cause during long-term follow-up^9,16^ This may account for why patients with an ischemic stroke are still at greater risk of mortality, in addition to stroke recurrence^17–20^, leading to direct death from cerebrovascular disease, that often occurs before stroke recurrence^21,22^ and also resulting in post-recurrence death^10,21,23^. It is therefore not sufficient to only evaluate the recurrence after first-time stroke without considering the risk of death competing with stroke recurrence and also the risk for death after recurrence. To do so, one of solutions is to divide the disease progress into three transition processes, from first-time stroke to cerebrovascular death, from first-time stroke to stroke recurrence, and from stroke recurrence to cerebrovascular death. Note that the two former transitions compete with each other and is supposed to show a reciprocal relationship. It should be also noted that the time to recurrence and the time to death after recurrence are also correlated. This means the duration of recurrence may also affect the survival after recurrence. To deal with these issues requires the use of time-dependent Cox regression mode, which can be used to compare the effect of two treatments, such as aspirin and nicametate, on three transitions rather than only one-jump process as shown in the previous study that found the aspirin group was less likely to have recurrence than the nicametate group. It is therefore possible that a lower rate of recurrence may be accompanied with a higher risk for the transition from first-time stroke to cerebrovascular death. Moreover, it requires a long-term follow-up study as if there is no long-term follow-up it is impossible to elucidate the relationship between time to recurrence and time to death from stroke as the pathway leading to death has been classified into death from stroke with and without recurrence. It is difficult to have such an evaluation for two treatments merely on the basis of short-term follow-up. The aim of this study is therefore to assess the respective effect of two treatments on stroke recurrence and cerebrovascular death with and without recurrence in first-time ischemic stroke patients with a long-term longitudinal follow-up cohort.

## Results

### Follow-up of the trial cohort

Of 466 patients, a total of 49 patients experienced stroke recurrence and the recurrence rate was 10.5%. The mean follow-up time for stroke recurrence was 14.04 months, and 21.03 months for non-recurrence. After 28 years of follow-up among the stroke recurrence patients, as of September 2019, 17 died of cerebrovascular disease, and 21 died of other competitive causes of death. Among the non-recurrent stroke, 72 died of cerebrovascular disease, and 200 died of other competitive causes of death. Cerebrovascular death, the major cause of death, accounted for 28.7% of all causes of death; diabetes and malignance accounted for 17.4% and 12.9% of all causes of death, separately.

Table 1 presents the baseline characteristics of study cohort by stroke statuses. Patients with stroke recurrence who had higher cerebrovascular death rate were age < 65 years, male and aspirin treatment, but not significant. The high total cholesterol level had a significantly lower cerebrovascular death rate. For patients without stroke recurrence, age < 65 years had a significantly lower cerebrovascular death rate.

**Table 1:** Baseline characteristics of patients based on stroke statuses.

Variables	Overall (N)	Stroke statuses							
		Total	Recurrence and CVD death	Recurrence and censored*	p-value	Total	CVD death without recurrence	Censored* without recurrence	p-value
		N	N (%)	N (%)		N	N (%)	N (%)	
**Age (y)**					0.54				0.02
< 65	250	23	9 (39.1)	14 (60.9)		227	30 (13.2)	197 (86.8)	
≧65	216	26	8 (30.8)	18 (69.2)		190	42 (22.1)	148 (77.9)	
Mean ± SD			64.6 ± 13.0	63.6 ± 11.8	0.78		65.9 ± 9.8	62.2 ± 11.1	< 0.01
**Sex**					0.72				0.20
Female	180	19	6 (31.6)	13 (68.4)		161	23 (14.3)	138 (85.7)	
Male	286	30	11 (36.7)	19 (63.3)		256	49 (19.1)	207 (80.9)	
**Treatment**					0.49				0.15
Nicametate	244	32	10 (31.3)	22 (68.7)		212	31 (14.6)	181 (85.4)	
Aspirin	222	17	7 (41.2)	10 (58.8)		205	41 (20.0)	164 (80.0)	
**Creatinine (mg/dl)**					0.94				0.24
≤ 1.4	413	43	15 (34.9)	28 (65.1)		370	61 (16.5)	309 (83.5)	
> 1.4	53	6	2 (33.3)	4 (66.7)		47	11 (23.4)	36 (76.6)	
**Glucose (mg/dl)**					0.57				0.91
< 100	170	17	5 (29.4)	12 (70.6)		153	26 (17.0)	127 (83.0)	
≧100	296	32	12 (37.5)	20 (62.5)		264	46 (17.4)	218 (82.6)	
**TG (mg/dl)**					0.23				0.26
< 150	218	23	6 (26.1)	17 (73.9)		195	38 (19.5)	157 (80.5)	
≧150	248	26	11 (42.3)	15 (57.7)		222	34 (15.3)	188 (84.7)	
**TC (mg/dl)**					0.06				0.95
< 200	195	20	10 (50.0)	10 (50.0)		175	30 (17.1)	145 (82.9)	
≧200	271	29	7 (24.1)	22 (75.9)		242	42 (17.4)	200 (82.6)	
**SBP (mmHg)**					0.92				0.45
< 140	92	9	3 (33.3)	6 (66.7)		83	12 (14.5)	71 (85.5)	
≧140	374	40	14 (35.0)	26 (35.0)		334	60 (18.0)	274 (82.0)	
Mean ± SD			156.6 ± 28.5	164.1 ± 30.6	0.42		159.3 ± 26.3	157.0 ± 26.3	0.51
**DBP (mmHg)**					0.95				0.31
< 90	172	17	6 (35.3)	11 (64.7)		155	23 (14.8)	132 (85.2)	
≧90	294	32	11 (34.4)	21 (65.6)		262	49 (18.7)	213 (81.3)	
Mean ± SD			94.8 ± 17.0	96.8 ± 17.9	0.71		92.4 ± 14.4	91.3 ± 14.6	0.56

*CVD* cerebrovascular disease, *TG* triglyceride, *TC* total cholesterol, *SBP* systolic blood pressure, *DBP* diastolic blood pressure.

*Non-cerebrovascular death is described as censored outcome.

### Effect of nicametate vs aspirin on recurrence and long-term survival

**Figure 1:**
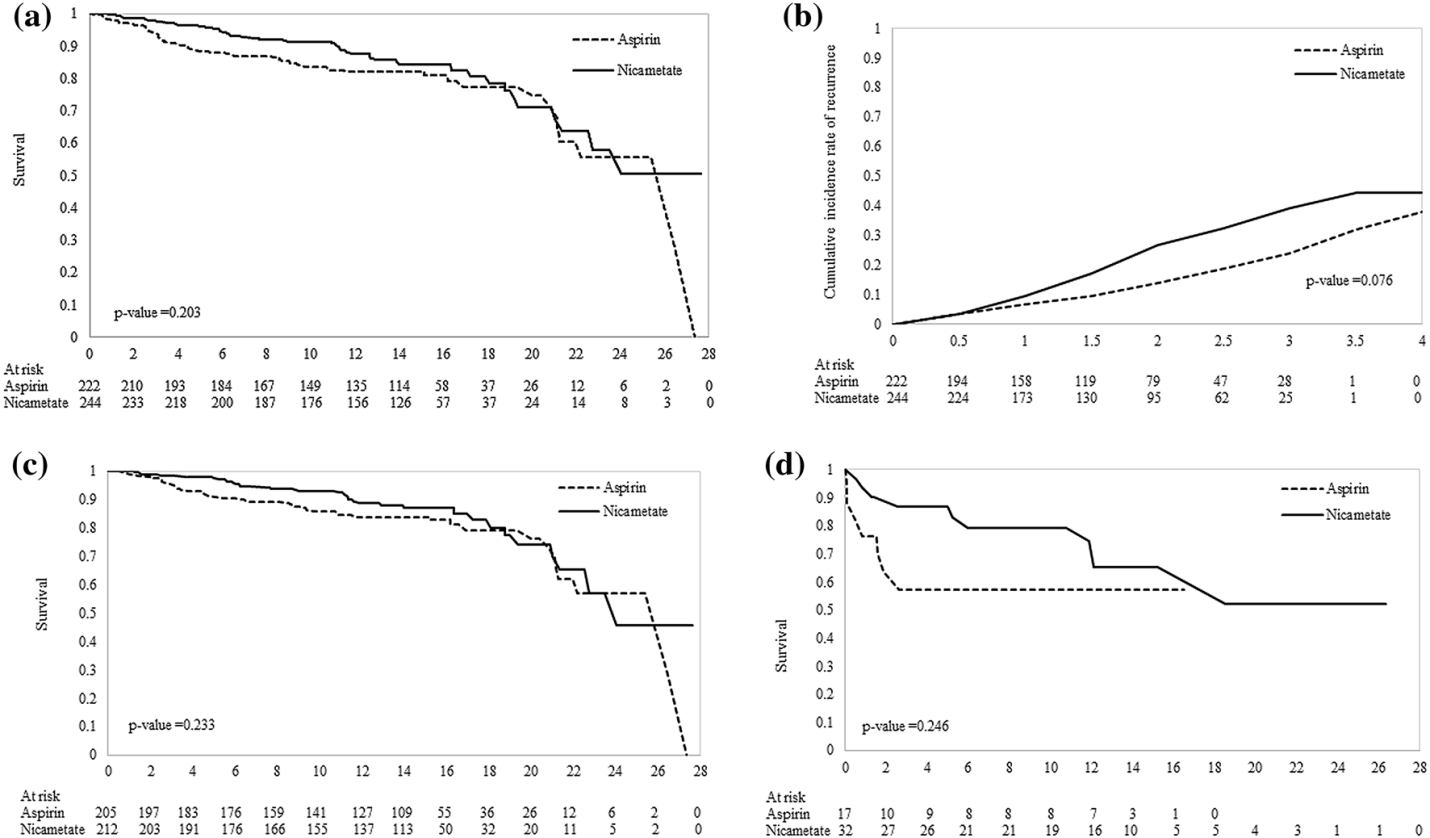
Cumulative survival and risk of recurrence by treatment groups. (**a**) Survival curve by treatment groups. (**b**) Cumulative recurrence rate by treatment groups. (**c**) Survival curve by treatment groups for patients without recurrent stroke. (**d**) Survival curve by treatment groups for patients with recurrent stroke.

Figure 1a–d show the overall long-term survival, recurrence, survival without recurrence, and the survival with recurrence. It is very interesting to note that the nicametate group had a higher recurrence whereas it had also higher survival rate regardless of whether recurrence was present although the difference was not statistically significant without using time-dependent Cox regression model.

Table 2 shows that the crude and adjusted hazard ratios (aHR) on the risk of cerebrovascular death for nicametate compared with aspirin. The multivariable time-dependent Cox model analysis revealed nicametate reduced the risk of cerebrovascular death about 37% (aHR: 0.63, 95% CI 0.41–0.97) compared with the conventional regiment of aspirin. Furthermore, stroke recurrence had higher risk for cerebrovascular death compared with non-recurrence patients (aHR: 3.01, 95% CI 1.73–5.23). Age ≧ 65 years has 2.26 times risk for cerebrovascular death compared with those age < 65 years (95% CI 1.46–3.51). Male has 1.66 times risk for cerebrovascular death compared with female (95% CI 1.04–2.63). Higher glucose level has 1.49 times risk for cerebrovascular death than glucose < 100 mg/dl (95% CI 0.95–2.32).

**Table 2:** Hazard ratios (HR) comparing nicametate with aspirin on cerebrovascular death in stroke patients using time-dependent Cox model.

	Univariate model ^a^			Multivariable model ^b^		
	Crude HR (95% CI)		p-value	Adjusted HR (95% CI)		p-value
**Treatment**						
Aspirin	1.00			1.00		
Nicametate	0.66	(0.41–1.02)	0.06	0.63	(0.41–0.97)	0.04
**Stroke recurrence**						
Non-recurrence	1.00			1.00		
Recurrence	2.53	(1.48–4.33)	< 0.01	3.01	(1.73–5.23)	< 0.01
**Age**						
< 65	1.00			1.00		
≧65	2.16	(1.40–3.32)	< 0.01	2.26	(1.46–3.51)	< 0.01
**Sex**						
Female	1.00			1.00		
Male	1.60	(1.02–2.51)	0.04	1.66	(1.04–2.63)	0.03
**Creatinine (mg/dl)**						
≤ 1.4	1.00			1.00		
> 1.4	1.78	(0.98–3.21)	0.06	1.52	(0.83–2.77)	0.18
**Glucose (mg/dl)**						
< 100	1.00			1.00		
≧100	1.26	(0.81–1.94)	0.31	1.49	(0.95–2.32)	0.08
**TG (mg/dl)**						
< 150	1.00			–		
≧150	0.85	(0.56–1.29)	0.44	–		
**TC (mg/dl)**						
< 200	1.00			–		
≧200	0.77	(0.52–1.17)	0.22	–		
**SBP (mmHg)**						
< 140	1.00			–		
≧140	1.00	(0.99–1.01)	0.77	–		
**DBP (mmHg)**						
< 90	1.00			–		
≧90	0.99	(0.98–1.01)	0.77	–		

^a^All univariate analysis incorporating recurrence as a time-dependent variable in the model.

^b^Variables control in multivariable analysis including age, sex, stroke recurrence and glucose level when we compared nicametate with aspirin.

## Discussion

In contrast to the original study addressing the effect of treatment on stroke recurrence^5^, we re-analyzed the data by assessing the effects of treatment on stroke recurrence and subsequent progression to cerebrovascular death by dividing the disease progress into three transition processes among ischemic stroke patients. Through such a thorough assessment, the efficacy of treatment in association with recurrence and the risk for death can be fully assessed. It is very interesting to see that while aspirin was associated with a lower risk of stroke recurrence nicametate reduced the competing risk of cerebrovascular death after first stroke and also reduced the risk of cerebrovascular death in the stroke subjects with recurrence. The benefits of aspirin have been shown to reduce the risk of stroke recurrence and all-cause mortality in previous studies^2,7^ but a recently study showed that use of aspirin was associated with a non-significant lower risk of cardiovascular disease based on competing risks consideration^28^. These findings were consistent with those noted in our results based on the use of duration-dependent Cox regression model and long-term survival of dying from cerebrovascular disease.

As a process of the pathologic change of vessel, previous studies have demonstrated the major role of endothelial dysfunction in the evolution of cerebrovascular disease^23,29,30^. The pharmacological effect of nicametate citrate on vasodilation provides a possible treatment in for peripheral vascular disease and cerebral ischemia. However, there is no decisive evidence regarding the efficacy of nicametate citrate on cerebrovascular disease in terms of recurrence and cerebrovascular death as we have shown here. Given its theoretical benefit for the circulation bed of cerebral system, it is thus worthwhile to validate such a long-term benefit by applying appropriate methods to take into account the process of ischemic stroke recurrence.

Regarding prognostic factors responsible for risk of cerebrovascular death, high glucose level (≧100 mg/dl) had an increased 1.49-time risk of cerebrovascular death. Previous studies indicated that elevated admission glucose or diabetes associated with increased risk of stroke recurrence and risk of death^31,32^. A recent study demonstrated that diabetes only had substantially increased risk of death following a first-time ischemic stroke in hemodialysis patients^33^. The difference might be due to the differences in study population and study design. Furthermore, higher Creatinine level (> 1.4 mg/dl) also had a higher risk of cerebrovascular death after stroke recurrence. Previous studies had reported that abnormal creatinine was associate with post-stroke mortality^34^ and increased risk of stroke^35^.

**Figure 2:**
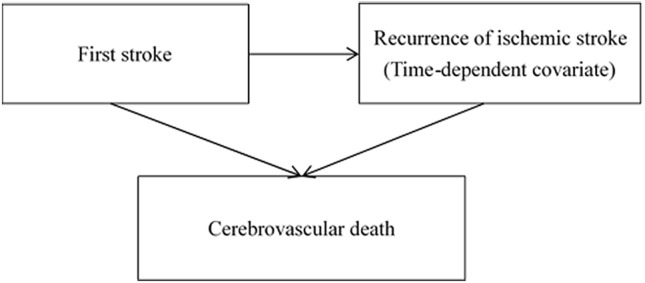
The disease progress process of stroke recurrence and cerebrovascular death during follow-up.

Stroke recurrence was a strong independent factor associated with cerebrovascular death with a threefold risk in our cohort. Previous studies have reported that 2.6 to 2.7-fold increased risk of all-cause mortality due to stroke recurrence^21,36^. Another study reported a 16.7-fold increase in the risk for death during 17-year follow-up^10^. Our findings that aspirin could reduce the recurrence of stroke but increased the risk of death from stroke may be reasonable. Generally speaking, the risk of hemorrhagic stroke is well acknowledged to increase in patients taking aspirin. Therefore, it is plausible that aspirin could reduce the risk of ischemic stroke recurrence while it could cause significantly more fatal hemorrhagic stroke^37,38^ compared with nicametate. However, as time to recurrence has been also included in the survival time from the date if first-time stroke, through recurrence, and then to death from cerebrovascular disease, it is not good not to take time to recurrence into account. This issue has been solved by using time-dependent Cox regression model by treating time to recurrence as time-dependent covariate as shown in Fig. 2. Note that occurrence of death may precede before recurrence. By using a time-dependent Cox model to consider the association between recurrence and death the influence of recurrence on all-cause mortality gave adjusted hazard ratio of 1.57 (92% CI 1.11–2.24) of adjusted hazard ratio compared with that of 2.68 (95% CI 1.55–4.64) when a time-dependent model was not applied in our study cohort.

**Figure 3:**
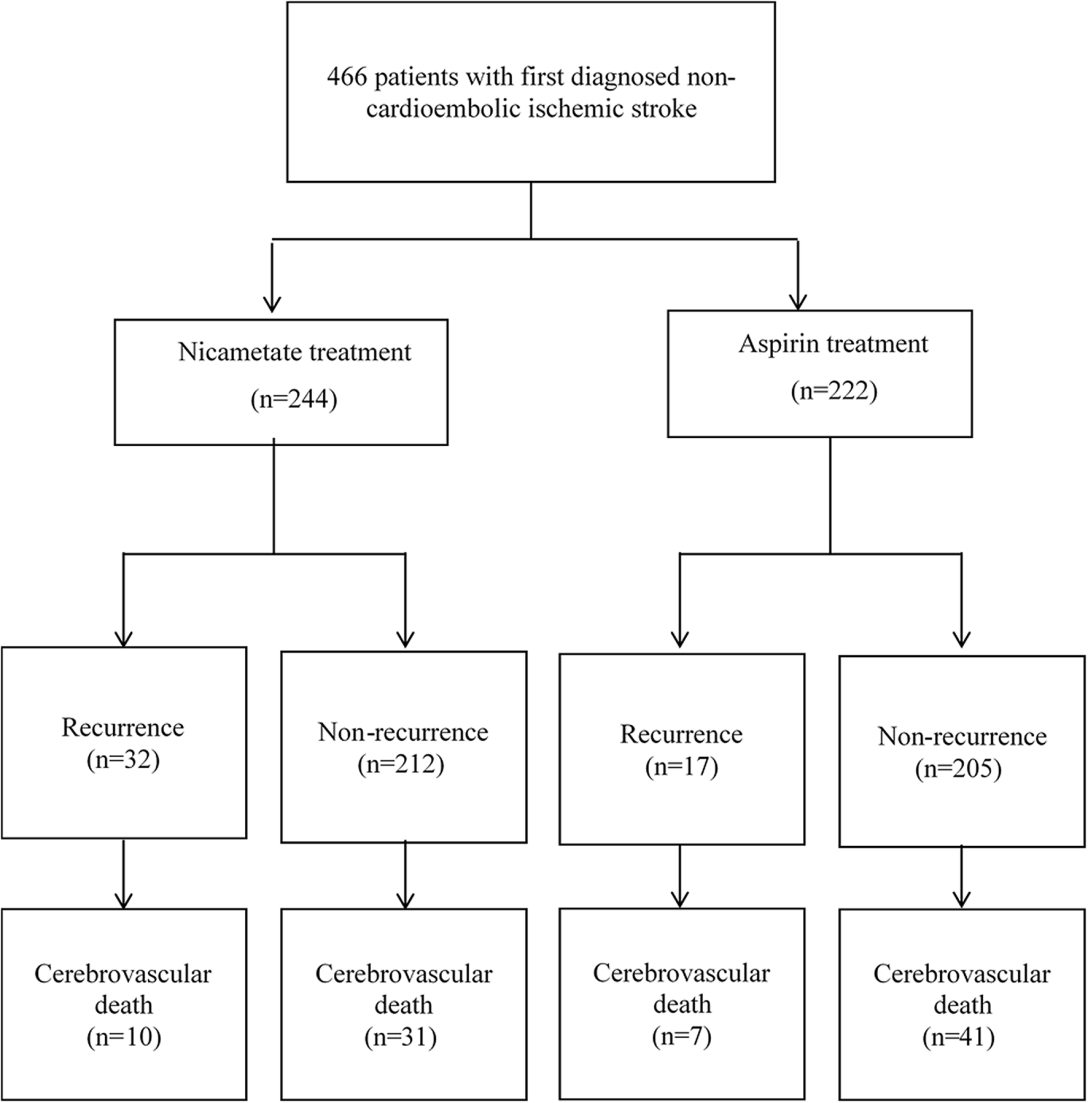
The follow-up of stoke recurrence and cerebrovascular death.

Given the substantial role of recurrence in the occurrence of cerebrovascular death, it is thus mandatory to consider its effect in assessing the treatment efficacy toward reducing the risk of cerebrovascular death. However, as illustrated in Fig. 3, cerebrovascular death can occur after first stroke of after the recurrence. The failure of taking into account the time-varying role of recurrence in efficacy assessment will diminish the treatment effect due to the error in calculating the time-to-cerebrovascular death interval by including time-to-occurrence period and the positive correlation between the two periods. Using recurrence as the terminal event to assess treatment efficacy will also result in misleading results since those benefit from the treatment may have a better survival and hence a higher chance for recurrence. To tackle this time-varying characteristic, we applied a time-dependent Cox regression model^24^ embedded in a disability model of stoke evolution. The usefulness of such a strategy in analyzing the evolution of terminal event with the consideration of the occurrence of accessory and intermediate pathway such as the recurrence in cerebrovascular death have been well demonstrated in previous studies^25–27^.

In light of our study findings aspirin still plays an important role in the reduction of recurrence in the first three years (high-risk period of recurrence) of follow-up since first stroke occurrence. One can consider combining medication with nicametate to prevent the death from cerebrovascular diseases. This implication and benefit should be investigated further using a large-scale long-term follow-up study to discuss the impact of various medications use in different periods but such a clinical application should be investigated in the future.

Our study has two limitations. First, the information on stroke recurrence was obtained within the period of clinical trial. It is possible that a patient may have experienced a recurrence or multiple recurrences after the end of trial. We may underestimate the effect of recurrence on cerebrovascular death. However, as the rate of stroke recurrence was higher in first two years of follow-up period, this may not substantially affect the final results. According to the previous studies from UK and Japan, they reported the proportions of 75%^11^ and 92%^39^ of those recurrent stoke patients who recurred within 2 years of follow-up since first stroke. Besides, there was 77% of recurrent stroke patients who recurred within first 3 years of follow-up among Chinese population^40^. Second, whether patients in the two arms continued to take medication after the end of trial or post stroke recurrence is unknown. However, this is a randomized controlled trial that health behaviors, awareness, or medication for control on blood pressure, glucose, or lipid, etc. would be balanced between two arms even after the trial.

Third, according to the mechanism and evidence-based researches, the treatment with aspirin for prevention on primary or secondary stroke may increase the risk of hemorrhagic stroke^37,38^. However, the cause of death ascertained by Health and Welfare Data Science Center (HWDC), in light of the regulation for open data use, only 26 and 36 brief classifications of cause of death were provided for before 2007 and 2008 onward, respectively (see Supplementary Table 1). Therefore, the further evaluation with outcomes of hemorrhagic or ischemic stroke are not incapable of analysis. So, the comparison for hemorrhagic or ischemic events between two drugs could not be evaluated. Further study with detail causes collection would be helpful to explore this issue. Forth, based on this existing study sample size with long-term follow-up, the power wore to less than 80% (power = 73%). However, it’s impossible to recruit additional subjects again, but it’s good experience for future study consideration on sample size estimation, especially considering long-term follow-up with high censoring rate.

In conclusion, the aspirin group had a lower recurrence rate than the nicametate group even with recurrence after 1–2 years of follow-up of first stroke but the latter had significantly reduced death from cerebrovascular disease for nicametate group, which requires more research to verify.

## Methods

### Data from a randomized controlled trial

Data used in this study were derived from a multicenter randomized double-blind controlled trial in Taiwan. The trial was designed to evaluate the effect of aspirin on prevention of stroke recurrence compared with nicametate among first-time noncardioembolic ischemic stroke patients and to identify what prognostic factors associated with stroke recurrence. The detail results of the trial and relative study have been published in elsewhere^5,15^. In brief, a total of 466 patients with first-time noncardioembolic ischemic stroke were enrolled from October 1992 through April 1995. Of them, 222 were randomly assigned to receive aspirin and 244 to receive nicametate.

Participants were required to have a stable stroke and to exclude might shorter anticipated survival (e.g., systemic disease, myocardial infarction within six weeks before the stroke, etc.). The eligible patients were randomly assigned to 50 mg nicametate citrate or 100 mg aspirin at 2 to 6 weeks after the onset of the first-time ischemic stroke. The primary outcome measure from the original trial was ischemic stroke recurrence. Ischemic stroke recurrence is defined as a neurological impairment lasting for more than 24 h in a new location or worsening of a previous neurological impairment lasting more than 7 days. Computed tomography scanning of brain or magnetic resonance imaging is administered in patient with stroke recurrence. Information and classification for stroke recurrence events were completely collected during the original trial period (Oct. 1992 – Apr. 1995) and had been published elsewhere by Lee et al^5^. Regarding information on death, both causes and date of death were linked by Taiwan National Death Registry System till Sep. 2019.

### Causes of death ascertainment

The Taiwan National Death Certification Registry System has been initialed since 1971 which covers all residents in Taiwan, mandated by regulation and law, and centralized governed by Ministry of Health and Welfare, Taiwan. According to the regulation, for those who died, death certificate should be issued within 7 days by physicians and registered to database within 30 days^41^. Those physicians and professional staff involved in certificate coding must be trained and audited by central office of National Death Certification Registry^42^. The cause of death was coded on the basis of International Classification of Diseases (ICD) version before 2008 and coded with 10th version since 2008 and onward. In 2003, our government also adopted the ACME (Automatic Classification of Medical Entry) system to standardize the procedure for ICD coding.

To facilitate the health-related databases in Taiwan for the wide spread use of research based the integrity and the confidentiality of electronic health record, the Health and Welfare Data Science Center (HWDC) and collaborative center was established by Division of Statistics, Ministry of Health and Welfare since 2011. All research projects and applications were issued by the committee of HWDC review board. Therefore, we ascertained the causes of death and the date of death from the HWDC using the unique personal ID, but all ID were de-identified as anonymous dataset for project analysis.

### Time-dependent recurrence and survival model design

In addition to elucidating the effect of two treatments on recurrence as designed in the primary study, the study design is also intended to study the respective effects on two transitions, first-time stroke to cerebrovascular death without recurrence and stroke recurrence to cerebrovascular death. Such a time-dependent recurrence and survival model design is illustrated in Fig. 2. To do so, the study cohort with the two arms has been followed to ascertain death information from the national death statistics register data until the end of September 2019. The source of death register data is from Health and Welfare Data Science Center. Cause of cerebrovascular death was based on ICD9-CM codes 430–438 or ICD10 codes I60-I69. The detail diagram of follow-up of stoke outcomes is presented on Fig. 3. Note that non- cerebrovascular death was regarded as censored cases that are not of main interest in the current study.

Clinical data included baseline demographics and clinical biochemical values included age, gender, types of treatment, creatinine, glucose, triglyceride, total cholesterol, systolic blood pressure and diastolic blood pressure. We defined cut-off points to present the dichotomous effect of variables on study analysis. High creatinine level is defined as creatinine > 1.4 mg/dl, high glucose level is defined as glucose ≧ 100 mg/dl, high triglyceride level is defined as triglyceride ≧ 150 mg/dl, high total cholesterol level is defined as total cholesterol ≧ 200 mg/dl , high systolic blood pressure (SBP) level is defined as SBP ≧ 140 mmHg and high diastolic blood pressure (DBP) level is defined as DBP ≧ 90 mmHg. The study was approved by the Institutional Review Board of National Taiwan University Hospital (No. of IRB:201908091RINC).

### Statistical analysis

Baseline characteristics were compared in univariate analyses based on stroke statuses: recurrence and cerebrovascular death. We use the imputation to deal with covariates with missing values in regression model by replacing missing values with respective mean values. All univariate analyses adjusted stroke recurrence as a time-dependent variable in the model to adjust the dependence of recurrence and mortality. A multivariable model was constructed by significant variables (p < 0.1) from the univariate models, as well as age and sex were always retained. The effect of stroke recurrence on cerebrovascular mortality was analyzed by Cox regression model with recurrence as a time-dependent covariate^24–27^. A cause-specific hazard regression model was applied to estimate hazard ratios, and death from other non-cerebrovascular disease was treated as competing risks. All analysis is conducted using SAS 9.4 statistical software.

### Sample size and statistical power calculation

Two parallel independent groups with equal sample size were employed to assess the efficacy of two treatments, 100 mg of ASA and 50 mg of nicametate, respectively. Given both the censoring rate (drop-out rate) and the event rate of the control group were 30% and 0.1506 derived from previous study, respectively^43^ and 1.5 years of subjects’ recruitment and 2 years of the follow-up period, respectively, the minimum sample size requirement for each group was 185 subjects given statistical power of 80% and 5% of alpha level^44^. Therefore, the priori power at the time of conduction had already reached 86.7% given the enrollment of 222 participants receiving aspirin. Although long-term follow-up may increase statistical power, but the statistical power might be attenuated by high censoring rate mainly resulting from non-cerebrovascular death. Following this idea, the statistical power based on 20 years of the extended follow-up and 74% censoring rate would be reduced to 73%, which may be slightly lower than 80% but can be still acceptable.

### Statement of informed consent

This study was based on the randomized controlled trial which was approved by Institutional Review Committee of National Taiwan University Hospital and supported by Ministry of Health and Welfare (project No: DOH-TD-019), then launched since Oct 1992. All enrolled subjects gave their informed consent. After 28 years, the passive long-term follow-up study using National database was proposed, which was reviewed and approved by Institute Review Board of National Taiwan University Hospital (No. of IRB:201908091RINC).

### Statement of human and animal rights

All procedures followed were in accordance with the ethical standards of the responsible committee on human experimentation (institutional and national) and with the Helsinki Declaration of 1975, as revised in 2008.

## Supplementary Information

Supplementary Information.
