# Supplementary material for: Impacts of treatments on recurrence and 28-year survival of ischemic stroke patients
Source: Sci Rep. 2021 Jul 27;11:15258. doi: 10.1038/s41598-021-94757-6 (PMC8316573; doi:10.1038/s41598-021-94757-6)
Supplement: Supplementary file 1 — Supplementary Information. [file 41598_2021_94757_MOESM1_ESM.pdf]

Supplementary Table 1.

Cause of death for open data use (issued by Health and Welfare Data Science Center (HWDC))

After 2008	Cause of death	ICD-10
01	Malignant neoplasms	C00-C97
02	Carcinoma in situ and Neoplasm	D00-D48
03	Anemias	D50-D64
04	Diabetes mellitus	E10-E14
05	Vascular dementia	F01-F03
06	Meningitis	G00, G03
07	Spinal muscular atrophy and related syndromes	G12
08	Parkinson's disease	G20-G21
09	Alzheimer's disease	G30
10	Hypertensive disease	I10-I15
11	Heart disease	I01-I02.0, I05-I09, I20-I25, I27, I30-I52
**12**	**Cerebrovascular disease**	**I60-I69**
13	Atherosclerosis	I70
14	Aortic aneurysm and dissection	I71
15	Influenza	J10-J11
16	Pneumonia	J12-J18
17	Acute bronchitis and bronchiolitis	J20-J21
18	Chronic lower respiratory diseases	J40-J47
19	Coal worker's pneumoconiosis	J60-J65
20	Airway disease due to specific/unspecific organic dust	J66, J68-J69
21	Ulcer of stomach and duodenum	K25-K28
22	Hernia and Paralytic ileus and intestinal obstruction without hernia	K40-K46, K56
23	Chronic liver disease and cirrhosis	K70, K73-K74
24	Cholelithiasis and Other diseases of gallbladder	K80-K82
25	Diseases of Skin and Subcutaneous Tissue	L00-L99
26	Diseases of the Musculoskeletal System and Connective Tissue	M00-M99
27	Nephritis, nephrotic syndrome and nephrosis	N00-N07, N17-N19, N25-N27
28	Pregnancy, Childbirth, and the Puerperium	O00-O99
29	Certain conditions originating in the perinatal period	P00-P96
30	Congenital malformations, deformations, and chromosomal abnormalities	Q00-Q99
31	Age-related physical debility	R54
32	Sudden infant death syndrome (SIDS)	R95
33	Accidents and adverse effects	V01-X59, Y85-Y86
34	Suicide	X60-X84, Y87.0
35	Homicide	X85-Y09, Y87.1
36	All other causes	

Before 2007	Causes of death	ICD-9-CM、ICD-8
01	Malignancies	140-208
02	Diabetes mellitus	250
03	Nutritional marasmus	261
04	Other and unspecified protein-calorie malnutrition	262-263
05	Anemias	280-285
06	Meningitis	320-322
07	Heart disease	390-392,393-398,410-414,420-429
08	Hypertensive disease	401-405
**09**	**Cerebrovascular diseases**	**430-438**
10	Atherosclerosis	440
11	Pneumonia	480-486
12	Influenza	487
13	Bronchitis、Emphysema、asthma	490-493
14	Ulcer of stomach and duodenum	531-533
15	Appendicitis	540-543
16	Chronic liver disease and cirrhosis	571
17	Nephritis, Nephritic Syndrome and Nephrosis	580-589
18	Hypertrophy of prostate	600
19	Abortion	630-639
20	Direct obstetric causes	640-646.651-676
21	Congenital anomalies	740-759
22	Certain conditions originating in the perinatal period	760-779
23	Accidents and adverse effects	800-949
24	Suicide	950-959
25	Homicide	960-969
26	All other causes	
